# How Do Indians Know?

**Published:** 1886-03

**Authors:** 


### HOW DO INDIANS KNOW ?

We have frequently in the course of our reading found stories of Indian medicine men and women, who absolutely cured disease by their knowledge of the malady with which the person on whom they operate were afflicted. In many cases it seemed a long continued practice of their old men and women with simple herbs that brought relief to the sick ; but there are cases that sometimes occur, and which are well proven, that go beyond anything that we can account for from the knowledge of the individual Indian medicine man, or any knowledge he may have of the human system. Is it intuition, or is it a more profound study of the nature of man that gives them the wonderful insight into the human system? Let it be what it may, there most certainly does exist among the American Indians, certain persons who, through some means, have become familiar with the human system, and sometimes operate wonderfully to produce most astonishing results. We have had a case reported which is well proved, and of which there can be no question. Let those who have lived among the Indians tell us how it is done.

The case of Mrs. J. C. Williston, of Cleveland, Ohio, is a very strange, although a perfectly reliable one. She has recently returned from an extensive trip after her most remarkable experience. Mrs. Williston is not yet thirty, but her hair is almost white, and her face bears the signs of a life of suffering. She has been the victim for years, at varying hours of night and day, of pains like the cutting of a knife, and physicians supposed her to be suffering from cancer of the stomach. Eminent physicians failed to exactly locate the trouble or afford the lady relief. She spent months in travel and large amounts of money endeavoring to find effective treatment for her malady, but most of the physicians whom she consulted said that her disease was cancer of the stomach and that death would ultimately result.

Last October she went to San Francisco, stopping and treating, while en route, at the soda springs of Idaho, but in vain. Later she tried the waters of Calestoga springs and the baths of Passo del Robles, without effect. San Francisco's best physicians could afford no relief, and she started for Sonora, Mexico, intending to visit some celebrated springs near Nogales. She was taken seriously ill at Tucson, Arizona. One day during her illness a Papago Indian, of local notoriety as a "medicine man," visited Tucson from St. Xavier's Mission. He was taken to Mrs. Williston's rooms and asked if he could tell her ailment. He looked at her, had her describe the pains and their location, and then with the exclamation, "Me sabe heap bad spirit," he rushed out and toward the mission. In a few hours he returned with herbs and a basket of mescal, a root used by the Indians for food.

He motioned to Mrs. Williston to swallow the herbs. They made her deathly sick, so much so that she almost died from fright, thinking she had been poisoned. The result, after a few hours, was the emission of a dead lizard that was fully four inches in length. It was apparently of a species common to the East, but how it had managed to live for so many years was the mystery. Mrs. Williston says that but one explanation occurs to her, which is, that when a child, and living at Philipsburg, N. J., she and her brother were accustomed to drink from a little brook that ran near the house. They would scoop the water up with their hands, and she thinks that possibly in this way she swallowed the embryo lizard. Mrs. Williston's recovery has been rapid, and she is now fairly on the way to a complete restoration to health. Though the taking of living objects into the system is not rare, medical men say that this is one of the most remarkable cases on record.

Had this lady died while suffering from the presence of that lizzard; it would always have been "cancer of the stomach," but a poor unlettered savage who could scarcely utter a word of English, made very short work of what the doctors, full of science and collegiate courses, pronounced a cancer, because they could think of nothing that it could be, if not cancer, and they will all dispute this important fact, but the woman will always remember the

"Poor Indian, whose untutored mind,
Sees God in clouds and hears him in the wind."

